# A Special Section of Reviews From BμG@S 2002: 1^st^ Symposium of the Wellcome Trust Funded Multi-Collaborative Microbial Pathogen Microarray Facility

**DOI:** 10.1002/cfg.200

**Published:** 2002-08


					Comparative and Functional Genomics
A Special section of reviews from BG@S 2002: 1st Symposium of the Wellcome Trust
Funded Multi-Collaborative Microbial Pathogen Microarray Facility
Edited by
Philip Butcher
Organising Committee
Philip Butcher Joseph Mangan Sarah Husain
Brendan Wren Keith Vass Ceridwen Davies
Neil Stoker Jason Hinds
The microbial pathogen microarray group, BG@S, was funded by The Wellcome Trust in
2001 under its Resources for Functional Genomics Initiative and is based at St. George's
Hospital Medical School in London, UK. It has over 30 collaborating groups around the UK
involved in both bacterial pathogenesis and data analysis. BG@S aims to construct whole
genome glass slide microarrays for 12 bacterial pathogens in 2 years, and to supply these, and
related training, to their collaborators for a further 3 years.
The meeting highlighted the collective progress made in the last year. Microarrays for
Mycobacterium tuberculosis, Campylobacter jejuni, Haemophilus influenzae and Yersinia
pestis, among others, are already in use and most of the talks described the use of these
arrays in comparative genomics and gene expression profiling studies. There was also a session
covering the analysis of data from microarrays, which has been identified as a bottleneck in
the use of microarrays by many groups.

				

